# *Drosophila* Insulin-Like Peptide 8 (DILP8) in Ovarian Follicle Cells Regulates Ovulation and Metabolism

**DOI:** 10.3389/fendo.2020.00461

**Published:** 2020-07-17

**Authors:** Sifang Liao, Dick R. Nässel

**Affiliations:** Department of Zoology, Stockholm University, Stockholm, Sweden

**Keywords:** insulin signaling, fecundity, relaxin, interorgan signaling, stress resistance

## Abstract

In *Drosophila melanogaster* eight insulin-like peptides (DILP1-8) are encoded on separate genes. These DILPs are characterized by unique spatial and temporal expression patterns during the lifecycle. Whereas, functions of several of the DILPs have been extensively investigated at different developmental stages, the role of DILP8 signaling is primarily known from larvae and pupae where it couples organ growth and developmental transitions. In adult female flies, a study showed that a specific set of neurons that express the DILP8 receptor, Lgr3, is involved in regulation of reproductive behavior. Here, we further investigated the expression of *dilp8*/DILP8 and *Lgr3* in adult female flies and the functional role of DILP8 signaling. The only site where we found both *dilp8* expression and DILP8 immunolabeling was in follicle cells around mature eggs. *Lgr3* expression was detected in numerous neurons in the brain and ventral nerve cord, a small set of peripheral neurons innervating the abdominal heart, as well as in a set of follicle cells close to the oviduct. Ovulation was affected in *dilp8* mutants as well as after *dilp8*-RNAi using *dilp8* and follicle cell Gal4 drivers. More eggs were retained in the ovaries and fewer were laid, indicating that DILP8 is important for ovulation. Our data suggest that DILP8 signals locally to *Lgr3* expressing follicle cells as well as systemically to *Lgr3* expressing efferent neurons in abdominal ganglia that innervate oviduct muscle. Thus, DILP8 may act at two targets to regulate ovulation: follicle cell rupture and oviduct contractions. Furthermore, we could show that manipulations of *dilp8* expression affect starvation resistance suggesting effects on metabolism. Possibly this reflects a feedback signaling between ovaries and the CNS that ensures nutrients for ovary development. In summary, it seems that DILP8 signaling in regulation of reproduction is an ancient function, conserved in relaxin signaling in mammals.

## Introduction

Eight insulin-like peptides (DILP1-8), encoded on separate genes, have been identified in *Drosophila melanogaster* (1–6). These are characterized by unique spatial and temporal expression patterns during the lifecycle [reviewed in (1, 5–7)]. Several of the DILPs, notably DILP2, 3, 5, and 6, have been extensively investigated in their roles in development, reproduction, metabolism and effects on lifespan [see (5, 7–11)]. The most recently discovered one, DILP8, acts on a relaxin receptor homolog Lgr3, which is a leucine-rich repeat containing G-protein coupled receptor (GPCR) (3, 4, 12–15). DILP8/Lgr3 have been studied in the larva where they are part of a circuit that regulates timing of development of adult progenitor cells in imaginal disk by controlling production of the steroid hormone ecdysone (Ecd) (3, 4, 12–14, 16, 17). DILP8 is produced in imaginal disk upon damage or tumor development and is released to act on Lgr3 in a small set of brain neurons that in turn inhibit release of prothoracicotropic hormone (PTTH) by acting on four lateral neurosecretory cells that innervate the prothoracic gland. This results in diminished production of Ecd and 20-hydroxy-Ecd (20E) (3, 4, 12–14, 16, 17). Thus, these studies show that when discs are damaged DILP8 signaling ensures that metamorphosis is delayed and growth of imaginal discs is slowed down, allowing regeneration of imaginal discs and symmetric growth of the adult organism. A role of *dilp8* in interorgan signaling has also been shown in early pupae upon increased Ras/JNK signaling (18). However, adult roles of DILP8 signaling are poorly known.

In adult female flies *dilp8* transcript seems to be primarily expressed in the ovaries and very low levels were detected in tissues of male flies (15) [see also FlyAtlas, Flyatlas.org (19) and FlyAtlas2, http://flyatlas.gla.ac.uk/FlyAtlas2/index.html, (20)]. Also the *Lrg3* expression is sexually dimorphic in the adult CNS (21). To our knowledge, only one study has explored the functional role of the DILP8/Lgr3 signaling system in adult flies. Thermogenetic activation of a subset of *Lgr3* expressing neurons in the abdominal ganglion reduced receptivity in reproductive behavior of females and decreased fecundity (21), suggesting a relaxin-like function of DILP8 in flies.

Here we undertook a study of the role of DILP8/Lgr3 in adult physiology, including fecundity. Utilizing *dilp8* mutant flies as well as the Gal4-UAS system (22) to manipulate levels of *dilp8* in a targeted fashion, we confirm a role in ovary function and ovulation, but also see effects on feeding and starvation resistance. We find that *dilp8*/DILP8 is expressed in follicle cells in egg chambers of ovaries in young flies, whereas *Lgr3* expression is primarily in neurons of the central and peripheral nervous systems, some of which are efferents that innervate oviduct muscle. Furthermore, a small number of follicle cells in the basal region of each egg chamber express the receptor. Thus, DILP8 participates in both interorgan and intraorgan signaling. The peptide seems to signal systemically from the ovaries to efferent neurons that in turn regulate follicle cell function and affect ovulation and fecundity. In addition, DILP8 acts more locally, in a paracrine fashion, on small sets of basal follicle cells. Taken together, our data support an evolutionarily conserved function of relaxin-like peptides in ovulation and fecundity.

## Materials and Methods

### Fly Stocks and Maintenance

The following fly strains (*Drosophila melanogaster*) were used in this study: *w*^1118^, Canton S, UAS-mCD8-GFP, w^*^, P{w[+mC]=UAS-GFP.S65T}, *R47A04*-Gal4 (FC2) (23), *Elav* gene-switch-Gal4 (Elav^*GS*^*-Gal4*) and w, lexAop-CD2 RFP; UAS-CD4-spGFP1-10, lexAop-CD4-spGFP11 were obtained from Bloomington *Drosophila* Stock Center (BDSC), Bloomington, IN, USA. Three *Lgr3*-Gal4 lines, *Lgr3*-Gal4::VP16 (III), R19B09-Gal4, *Lgr3*-Gal4::p65/TM3,Sb, and a UAS-line, JFRC81-10XUAS-Syn21-IVS-GFP-p10 (21, 24), were obtained from Michael Texada (Janelia Farm, Ashburn, VA, USA). An *engrailed* (*en*)-Gal4 (25) was provided by Dr. Vasilios Tsarohas (Stockholm, Sweden); *dilp8*^*MI*00727^ (*dilp8* mutant with enhanced green fluorescent protein (eGFP) trap in the gene's first intron [Mi{MIC}CG14059MI00727]), *dilp8*-Gal4 and UAS-*dilp8*::3xFLAG (4) were from Maria Dominguez (Alicante, Spain); *dilp2*-Gal4 (8) from E. Rulifson (Stanford, CA); *ppl*-Gal4 (26) from M.J. Pankratz (Bonn, Germany), *c929*-Gal4 (27) from Paul H. Taghert (St Louis, MO); and UAS-*dilp8*/TM3,Sb (3) was provided by P. Leopold (Nice, France) and w^1118^; daughterless-GeneSwitch-Gal4 (28) was from V. Monnier (Gif-sur-Yvette, France). A *dilp2*-lexA::VP16 (29) was obtained from Zhefeng Gong (Hangzhou, China), forwarded by Annick Sawala and Alex Gould (London, UK). A double balancer Sco/Cyo,dfd-GFP; Dr/TM6bTb,dfd-GFP was used to combine dilp2-lexA::VP16 with R19B09-Gal4 for GRASP analysis.

Fly stocks were reared and maintained on standard Bloomington medium (https://bdsc.indiana.edu/information/recipes/bloomfood.html) at 18°C. The experimental flies were kept at 25°C on an agar-based diet with 10% sugar and 5% dry yeast. To activate *Elav*^*GS*^-Gal4 in adult stage, flies were fed RU486 (mifepristone; Sigma, St. Louis, MO, USA) dissolved in ethanol and added to the food at a final concentration of 20 μM. The same amount of ethanol was added to the control food.

### Production of DILP8 Antisera

To generate of DILP8 antisera we selected two sequences from the *D. melanogaster* DILP8 protein for custom synthesis:

DmDILP8_42−60_: NH_2_-CEHLFQADEGARRDRRSIE-CONH_2_ (19 aa)
DmDILP8_70−84_: NH_2_-CGSGKTHNKHHYISRS-CONH_2_ (16 aa)

These were coupled to thyroglobulin at the N-terminal cysteine (C), and the two peptide conjugates were mixed and injected as a cocktail into two rabbits for 3 months of immunization. The production of the antigens and the immunization were performed by Pineda Antibody Service (Berlin, Germany). The antisera were affinity purified before use. Preimmune sera did not immunolabel follicle cells when applied at 1:10,000.

### Immunocytochemistry and Imaging

Immunohistochemistry of *Drosophila* larval and adult tissues were performed as in previous study (30). Tissues were dissected in phosphate-buffered saline (PBS) and then fixed in 4% ice-cold paraformaldehyde (PFA) (2 h for larval samples and 4 h for adults), and subsequently rinsed in PBS for 1 h. Samples were then incubated for 48 h at 4°C in primary antibodies diluted in PBS with 0.5% Triton X-100 (PBST). After washes in PBST for 1 h at room temperature, the samples were incubated with secondary antiserum for 48 h at 4°C. Finally, all samples were washed with PBST and PBS, and then mounted in 80% glycerol with 0.1 M PBS. Images were captured with a Zeiss LSM 780 confocal microscope (Jena, Germany) using 10 ×, 20 ×, or 40 × oil immersion objectives. Images of the whole fly were captured with a Zeiss Axioplan 2 microscope after freezing the flies at −20°C. Confocal and microscope images were processed Fiji (https://imagej.nih.gov/ij/) and for contrast and intensity in Adobe Photoshop.

The following primary antisera were used: rabbit anti-DILP8 (this study) used at 1:10,000; mouse anti-green fluorescent protein (GFP) at 1:000 (RRID:AB_221568, Invitrogen, Carlsbad, CA). Rabbit anti-leucokinin (31) at 1:2,000; rabbit anti-ion transport peptide (32) at 1:1,500; rabbit anti-DILP3 (33) at 1:2,000 provided by J. A. Veenstra, Bordeaux, France, rabbit anti-tyrosine decarboxylase-2 (Tdc2; pab0822-P, Covalab, Cambridge, UK; at 1:200 [see (34)] obtained from D. Pauls, Leipzig, Germany; Rhodamine-phalloidin was used at 1:1,000 to stain muscle. The following secondary antisera were used: goat anti-rabbit Alexa 546, goat anti-rabbit Alexa 488 and goat anti-mouse Alexa 488 (all from Invitrogen). DAPI (Sigma) at a dilution of 1:2,000 was used to staining the nuclei.

### Starvation Resistance Experiments

Six to seven days old female flies were used for starvation resistance experiments. The flies were placed in vials containing 0.5% aqueous agarose for starvation in an incubator at 25°C with 12:12 h Light:Dark (LD) conditions and controlled humidity. Dead flies were counted at least every 12 h. For desiccation resistance the experiment was the same, except that flies were kept in empty vials, thus the flies obtained no food and no water.

### Determination of Eclosion Time

To determine the eclosion time, 1-week-old adult parental flies were mated in the evening. The next morning these mated flies were transferred to vials with fresh food for egg laying during 4 h. After that the adult flies were removed. Two hours after the initiation of oviposition was considered time “0,” and thereafter, the number of flies eclosed from these vials were monitored at least every 12 h.

### Oviposition and Egg Numbers in Ovaries of the Flies

For oviposition, 5 or 7 days old flies were used. The number of eggs laid by individual pairs of flies was counted every 24 h for 2 days, and the total number of eggs laid in 48 h was calculated. After the egg laying experiments, the same flies were used to monitor the number of eggs that are at stage 10–14 [according to (35–37)] inside each female fly.

### Statistical Analysis

Prism version 6.00 (La Jolla, CA, USA) was used for statistics and generating the graphs. The experimental data are presented as means ± SEM. Data were checked firstly with Shapiro-Wilk normality test, then one-way analysis of variance (ANOVA) was used for comparisons among three groups or Student's *t*-test was performed followed with Tukey's multiple comparisons test when comparing two groups. Survival data were compared with survival analysis (Log Rank comparison with Mantel-Cox post test).

## Results

### Distribution of dilp8/DILP8

**Figure 1:**
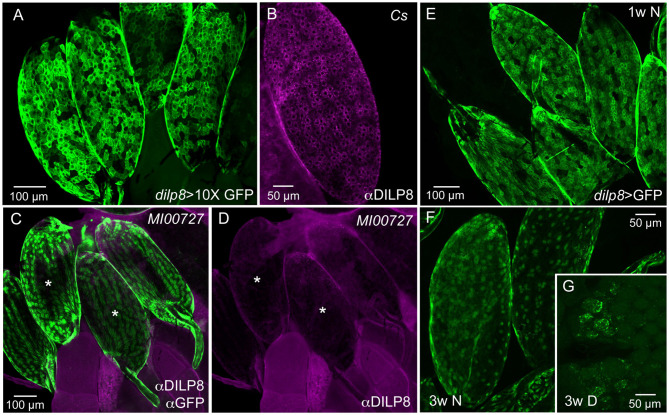
Ovarian follicle cells of mature eggs express dilp8/DILP8. **(A)** Using a 10X-GFP the dilp8-Gal4 expression is strong in follicle cells surrounding mature eggs (stage 14). **(B)** DILP8 antiserum labels follicle cells in mature eggs (stage 14) in a wild type fly (Cs, Canton s). **(C)** A dilp8 mutant fly (dilp8^MI00727^) with an eGFP-insertion reveals GFP expression in follicle cells. The sample was also incubated in anti-DILP8. **(D)** DILP8 antiserum yields no immunolabeling in the same specimen as in **(C)**, due to the dilp8 mutation. Asterisks label the same two eggs in **(C,D)**. **(E)** In 1-week-old flies kept under normal conditions dilp8-Gal4 expression (dilp8 > GFP) is seen in follicle cells of stage 14 eggs [in **(E–G)** 10X-GFP was not used]. **(F)** In 3-week-old flies dilp8-Gal4 expression in mature eggs is somewhat weaker. **(G)** In flies kept for 3 weeks in reproductive diapause ovary development is arrested and no eggs develop into vitellogenic stages and only residual dilp8-Gal4 expression is seen in unidentified cells.

The distribution of DILP8 producing cells was monitored with the Gal4-UAS system (22) using a *dilp8*-Gal4 line to drive GFP (Figure 1A). We also employed immunocytochemistry with a novel antiserum to DILP8 (Figure 1B) and a *dilp8* mutant with enhanced green fluorescent protein (eGFP) trap in the gene's first intron, *dilp8*^*MI*00727^ (4) (Figure 1C). Tissue expression data indicates that in adults the *dilp8* transcript is high in females, where it is seen predominantly in ovaries, whereas male expression is overall very low [modENCODE_mRNA-Seq_tissues, (38); http://flyatlas.gla.ac.uk/FlyAtlas2/index.html, (20)]. Thus, we analyzed only females and observed *dilp8*-GFP in follicle cells in egg chambers of ovaries in 1–7 d old flies (Figure 1A). The *dilp8*-Gal4 expression was only detected in mature eggs (stage 14) where it was seen in most follicle cells with no obvious regional differences (Figure 1A). DILP8 immunolabeling was also detected in follicle cells, and again was only seen in mature eggs (Figure 1B). To control for antiserum-specificity we used *dilp8* mutant flies (*dilp8*^*MI*00727^) with an eGFP insertion and could show that the DILP8 antiserum no longer recognized the GFP-labeled follicle cells (Figures 1C,D). As another control for the specificity of the anti-DILP8 we show here that it labels punctates in wing imaginal disk after induction of disrupted development [see (3, 4)] by knockdown of syntaxin 7 (*avalanche; avl*) through driving *avl*-RNAi with an *egrailed*-Gal4 driver (Supplementary Figure 1).

The *dilp8-*GFP expression (*dilp8*>GFP) in follicle cells is prominent in mature eggs of young flies, but can still be seen in 3-week-old ones (Figures 1E,F). In flies kept for 3 weeks in reproductive diapause, sparse *dilp8*-GFP is seen in cells of the rudimentary ovaries, but since mature eggs are absent no labeled follicle cells were detected (Figure 1G). Congruent with this, a decrease in *dilp8* expression during adult diapause was shown earlier in a genome-wide analysis of gene transcripts (39).

We could not detect DILP8 immunolabeling outside the ovaries in adult flies under normal conditions. However, *dilp8*-Gal4 expression could be seen in cells of the hindgut and the salivary glands (Supplementary Figures 2A,B). The gut expression is supported by single cell transcriptomics data where *dilp8* was found in intestinal muscle cells and enteroblasts [http://flygutseq.buchonlab.com/data; (40)]. In third instar larvae, no DILP8 immunolabeling was seen unless imaginal discs were damaged (Supplementary Figure 1), but *dilp8*-GFP was detected in neuronal progenitors in the brain (Supplementary Figures 2C,D). Thus, under normal conditions weak DILP8 immunolabeling only appears in follicle cells of mature eggs (stage 14) and we additionally provide two lines of evidence for *dilp8* expression in these cells at this stage, the use of *dilp8*-Gal4>UAS-GFP and the *dilp8*^*MI*00727^ line with an eGFP insertion. Importantly, it was also shown by single-cell transcriptome sequencing that *dilp8* transcript displays a peak expression in follicle cells of late stage 14 eggs (41).

### Distribution of the DILP8 Receptor Lgr3

**Figure 2:**
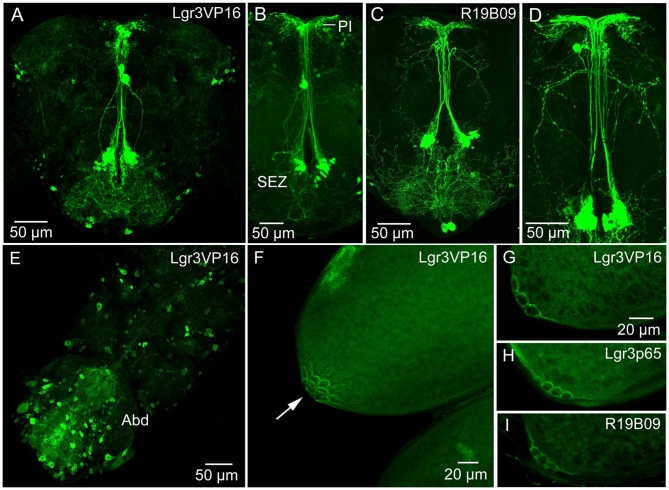
Expression of Lgr3-Gal4 in the CNS and ovaries. **(A)** Lgr3-Gal4 expression in neurons of the adult brain using the Lgr3VP16-Gal4 driver. **(B)** The same driver in a different brain showing neurons of the subesophageal zone (SEZ) that supply processes to the pars intercerebralis. **(C,D)** Another Lgr3-Gal4 driver (R19B09) displays a similar expression. **(E)** Numerous neurons in the adult ventral nerve cord express Lgr3-Gal4, especially in the abdominal neuromeres (Abd). **(F)** Lgr3-Gal4 expression in posteriorly located follicle cells (arrow). This figure shows a Z-stack of optical sections. **(G–I)** Lgr3-Gal4 expression in follicle cells seen with the three Gal4 drivers used in this study. These figures show 2–3 optic sections.

The widespread distribution of the DILP8 receptor, Lgr3, has been described by Gal4-GFP expression in the central nervous system (CNS) of larvae (12–14, 16) and adults (21). These studies show numerous *Lgr3*-Gal4 expressing neurons in the CNS. It should be noted that there is no independent evidence that the *Lgr3*-Gal4 neurons express the receptor (no antiserum available yet). We used three *Lgr3*-Gal4 lines and could confirm the general distribution in the brain and ventral nerve cord of adults (Figures 2A–E) shown by (21). Although there are some differences in the expression patterns of the three lines, some brain neurons were seen in all. A set of brain *Lgr3-*Gal4 neurons have processes in the pars intercerebralis (PI) and tritocerebrum/subesophageal zone (Figures 2A–D) where also insulin producing cells (IPCs) and other median neurosecretory cells (MNCs) arborize [see (42, 43)]. To test whether the *Lgr3* expressing neurons are in synaptic contact with DILP2,3,5-producing IPCs we employed GFP-reconstitution across synaptic partners (GRASP) technique (44, 45). Using a R19B09-Gal4 and a *dilp2*-LexA line as well as UAS-spGFP1-10 and LexAop-spGFP11 we could show that in the larval brain IPCs are connected to *Lgr3*-Gal4 expressing neurons (Supplementary Figures 3A,B). However, in the adult brain we found no reconstituted GFP (Supplementary Figures 3C,D) suggesting the neuron types are no longer connected.

We examined Lgr3 expression also in the CNS of larvae (Supplementary Figures 4A,B). A set of 14 *Lgr3*-Gal4 expressing neurons in the larval abdominal neuromeres co-express the neuropeptide leucokinin (LK) (Supplementary Figures 4A,B). In adults, these neurons have been shown to use LK to regulate secretion in Malpighian tubules in control of ion and water homeostasis [(46); see also (47)]. However, we could not detect colocalized Lgr3 and LK in these cells in adults.

Importantly, using all three Gal4 lines, we found *Lgr3*-Gal4 expression in a small subset of follicle cells of mature eggs (stage 14) (Figures 2F–I). These follicle cells are located closest to the oviduct, in the same position as cells previously found to express the Matrix metalloproteinase 2 (Mmp2), that was shown to be required for ovulation (48), Furthermore, we detected *Lgr3* expression in a small set of peripheral bipolar neurons that innervate the dorsal aorta (heart) in the abdomen (Supplementary Figures 4C–H). A median pair of such *Lgr3*-expressing aorta-associated cells co-express ion transport peptide (ITP) (Supplementary Figures 4E–H). Similar median cells were identified by ITP antiserum several years ago (49).

**Figure 3:**
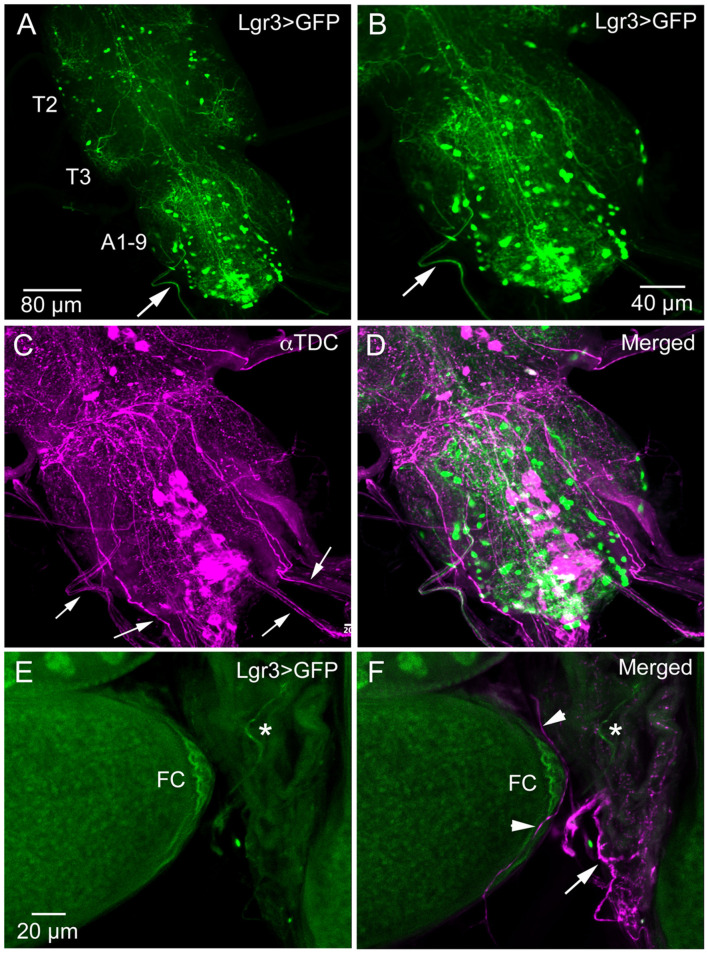
Lgr3-Gal4 and octopamine expressing efferent neurons innervate oviduct muscle. **(A)** Overview of adult thoracic (T2, T3) and abdominal (A1-9) neuromeres with Lgr3-Gal4 expressing neurons. One efferent axon emerging from the abdominal neuromeres is seen at arrow (Lgr3VP16-Gal4). **(B–D)** The same specimen, which has also been immunolabeled with antiserum to Tdc2 (tyrosine decarboxylase; magenta). Note that the peripheral Lgr3-expressing axon at large arrows does not colocalize Tdc2 and Lgr3 labeling. Also note that quite a few Tdc2 immunolabeled peripheral axons emerge from the ventral nerve cord (small arrows), some probably destined for the oviduct muscle. **(E,F)** Part of the ovary and oviduct muscle with Lgr3 (Lgr3VP16-Gal4) and Tdc2 expression. Basal follicle cells (FC) express Lgr3-Gal4 and so does one axon on oviduct muscle (at asterisk). In **(F)** several axons immunolabeled with anti-Tdc2 are seen on oviduct muscle (arrow and arrow heads). No colocalization of Lgr3 and Tdc2 could be detected.

We did not try to analyze the many *Lgr3*-Gal4 expressing interneurons in the ventral nerve cord (and the three Gal4 drivers displayed differences in expression pattern). However, looking at the *Lgr3*-expressing neurons of the abdominal neuromeres in more detail we detected axons running toward the periphery (Figures 3A–D). Since it is known that the ovaries and oviduct are innervated by octopaminergic neurons (34, 50, 51), we performed double labeling with *Lgr3*-Gal4-driven GFP and immunostaining with antiserum to Tdc2, a biosynthetic enzyme catalyzing the production of octopamine (OA) and tyramine (Tyr). We detected Tdc2-immunlabeled axons running posteriorly from abdominal neuromeres (Figures 3C,D), but found no clear evidence for co-expression of *Lgr3* and Tdc2 (Figure 3D). When analyzing the expression in the ovaries and oviduct, we found separate *Lgr3-Gal4* and Tdc2 expressing axons and no colocalization (Figures 3E,F). Thus, we identified some *Lgr3*-expressing axons innervating oviduct muscle, but have no evidence that these *Lgr3* neurons also produce OA or Tyr.

### Functional Roles of DILP8 Signaling

**Figure 4:**
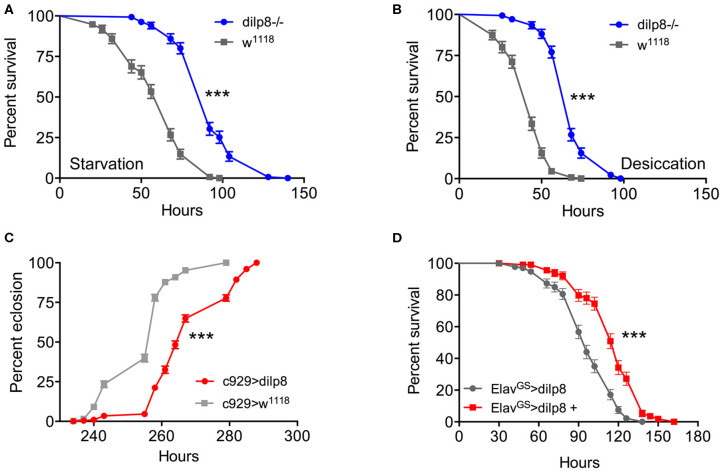
Manipulations of dilp8 affect stress responses and developmental time. **(A,B)** The dilp8 mutant flies survive starvation and desiccation better than controls. We used 135 flies per genotype from three independent replicates [***p < 0.001, as assessed by log-rank (Mantel–Cox) test]. **(C)** Overexpression of dilp8 with the c929-Gal4 driver delays adult eclosion (time from egg to eclosion). The eclosion time of more than 394 flies were analyzed here, [***p < 0.001, as assessed by log-rank (Mantel–Cox) test]. **(D)** Using a drug-inducible pan-neuronal driver, gene-switch Elav-Gal4 (Elav^*GS*^-Gal4) we could induce dilp8 in 5-day-old flies by feeding RU486 (Elav^*GS*^-Gal4 > dilp8+). Ectopic dilp8 increases starvation resistance. More than 96 flies from three independent replicates were used [***p < 0.001, as assessed by log-rank (Mantel–Cox) test]. See also Supplementary Figure 6C for another conditional experiment.

Next, we addressed the functional roles of DILP8 signaling in adults. Since several of the DILPs are known to affect stress responses (2, 5, 9, 52), we explored the role of *dilp8* in this respect. Using the *dilp8* mutant flies (*dilp8*^*MI*00727^), we tested the effect loss of *dilp8* on resistance to starvation (Figure 4A) and desiccation (Figure 4B). In both tests the mutant flies survived longer than control flies. To analyze the effect of *dilp8* gain of function we used different Gal4 lines to drive UAS-*dilp8*. The efficacy of these lines is shown in Supplementary Figure 5. We used a Gal4 line *c929* that drives expression in several hundred peptidergic neurons characterized by the transcription factor *Dimmed* (27). With the *c929*-Gal4 a substantial number of *Dimmed* neurons could be labeled with antiserum to DILP8 (Supplementary Figures 5A,B). It can be noted that *dilp8* overexpression leads to a down-regulation of DILP3 expression in insulin producing cells (IPCs) of the brain (Supplementary Figures 5C–E) and possibly affects other DILPs (not tested). Also a *dilp2*-Gal4 is able to induce DILP8 immunolabeling in IPCs (Supplementary Figure 5F). We also used gene-switch Gal4 drivers (*Elav*^*GS*^ and *daughterless*^*GS*^-Gal4), where the Gal4 is activated by feeding the flies RU486 (mifepristone), to switch on DILP8 in neurons of the adult fly (Supplementary Figures 5G–K).

As seen in Figure 4C, over-expression of *dilp8* with the *c929*-Gal4 delays adult eclosion (time from egg to adult eclosion increases). This is as expected from the role of *dilp8* in inhibiting Ecd production and thereby causing developmental delay (3, 4). Both *c929*- and *dilp2*-Gal4 driven *dilp8* results in increased survival during starvation (Supplementary Figure 6A). We next employed a driver, *pumpless* (*ppl*) that directs expression to the fat body. Using *ppl* > *dilp8* we noted that 1-week-old flies survived starvation better than controls (Supplementary Figure 6B). To test the temporally restricted effect of *dilp8* overexpression in adult flies, we employed *Elav*^*GS*^-Gal4 flies and activated by feeding 1-week-old flies RU486 and found that flies display increased starvation resistance (Figure 4D). We furthermore tested broad ectopic *dilp8* expression driven with a *daughterless*^*GS*^-Gal4 and obtained a similar adult-specific result (Supplementary Figure 6C). These conditional experiments confirm that broad ectopic *dilp8* expression increases starvation resistance in adults. The effects of ectopic *dilp8* expression on survival during starvation are surprisingly the same as seen in *dilp8* mutant flies. In the *dilp8* mutant the effects might reflect the loss of action of endogeneous DILP8 on its receptor. However, it is possible that the ectopic DILP8 may act not only on the GPCR Lgr3, but also on the insulin receptor, dInR (a receptor tyrosine kinase), similar to the other *Drosophila* relaxin-like peptide, DILP7 (53, 54). It was actually shown that dInR could be captured by DILP8 in a ligand capture assay, suggesting that DILP8 is able bind to both Lgr3 and dInR (13). Hence, DILP8 could act on tissues that are not normally targeteted by the peptide and result in gain of function phenotypes.

**Figure 5:**
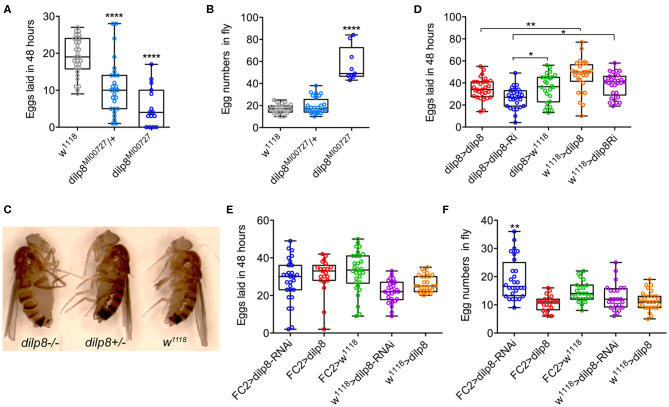
Manipulations of dilp8 affect fecundity in flies. **(A)** dilp8 mutants (hetero- and homozygous) lay fewer eggs than controls. Fifteen to thirty flies from three replicates were used (****p < = 0.00001, one-way ANOVA followed by Tukey's test). **(B)** The number of eggs retained in the flies is significantly higher in homozygous dilp8 mutants. Nine to twenty-seven flies from three replicates were used (****p < 0.0001, one-way ANOVA followed by Tukey's test). **(C)** The higher number of eggs retained in dilp8 mutant flies is reflected in the bloated abdomen (not seen in heterozygote or control). **(D)** Knockdown of dilp8 using dilp8-Gal4 significantly reduces egg laying over 48 h, whereas dilp8 overexpression has no effect (*p < 0.05, **p < 0.01). **(E)** Overexpression and knockdown of dilp8 in follicle cells (FC2-Gal4) does not significantly affect egg laying over 48 h. Twenty-one to thirty flies from three replicates were used (one-way ANOVA followed by Tukey's test). **(F)** Knockdown of dilp8 in follicle cells leads to a higher number of eggs retained in flies, but overexpression has no effect [20–28 flies from three replicates were used (**p < 0.01, one-way ANOVA followed by Tukey's test)].

Inspired by the expression of *dilp8*/DILP8 in follicle cells and *Lgr3* in a subset of follicle cells in the basal part of the egg chamber, as well as in axons innervating the oviduct, we went on to test the role of *dilp8* in fecundity. The number of eggs laid is significantly diminished in *dilp8* mutant flies (Figure 5A). This reduced number appears to be a result of defects in ovulation since the number of eggs retained in the ovaries is higher in *dilp8* mutant flies (Figures 5B,C). As seen in Figure 5C, the increased number of eggs in ovaries is reflected in an enlarged abdomen in the mutant flies. We also overexpressed and knocked down *dilp8* with a *dilp8*-Gal4 driver, but only noted a significant reduction of eggs laid for *dilp8* > *dilp8*-RNAi (Figure 5D). Next, we tested the effects of manipulating *dilp8* levels specifically in follicle cells by overexpression or knockdown with the *FC2*-Gal4 driver (23) (Figures 5E,F). These manipulations did not significantly affect the number of eggs laid in 48 h, but the number of eggs remaining in the ovaries increased after *dilp8*-RNAi in follicle cells (Figure 5F). Thus, *dilp8* in follicle cells appears critical for ovulation.

## Discussion

**Figure 6:**
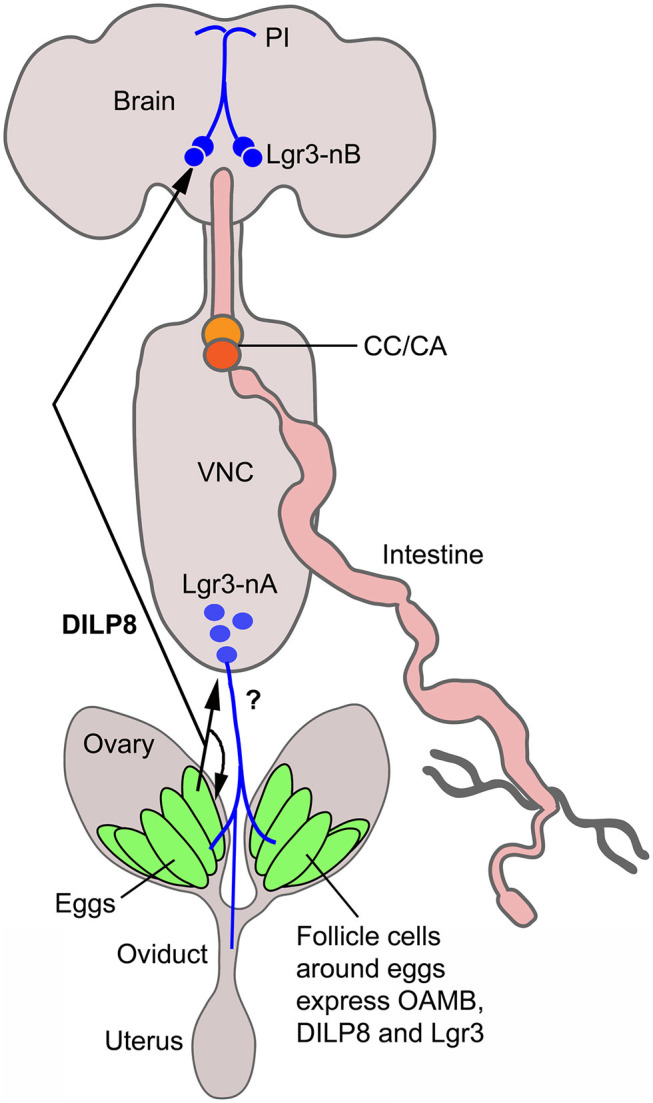
Summary diagram of DILP8 signaling in adult Drosophila. DILP8 is produced in follicle cells of the ovaries and can act on the receptor Lgr3 expressed on neurons of the CNS (blue) and on follicle cells near the oviduct. The efferent neurons Lgr3-nA in the ventral nerve cord (VNC) innervate the oviduct, but we found no evidence for octopamine (OA) coexpression, so the neurotransmitter is unknown (?). Other efferent neurons have been shown to produce OA and regulate ovulation via the OA receptor OAMB. A set of Lgr3 neurons (Lgr3-nB) in the brain supplies processes to the pars intercerebralis (PI) where IPCs and other median neurosecretory cells (MNCs) have dendrites. It is tempting to speculate that DILP8 acts on such neurons and that these in turn regulate release of DILPs or other peptide hormones in MNCs to regulate feeding and metabolism. CC/CA, corpora cardiaca/corpora allata.

Our study suggests that *dilp8* in follicle cells of ovaries is important for fecundity in *Drosophila*. We could show *dilp8*-Gal4 expression and DILP8 immunolabeling in follicle cells in mature eggs of young mated and unmated flies. Flies mutant in *dilp8* lay fewer eggs, but seem to produce normal numbers of eggs that are retained within the ovaries, suggesting effects on ovulation. Knockdown of *dilp8* specifically in follicle cells also resulted in flies with more eggs retained in the ovaries, (but we did not see fewer eggs laid). Since we could not detect both *dilp8* and DILP8 expression outside the ovaries in adult flies under normal conditions, we suggest that follicle cells are a primary source of the peptide hormone in regulation of fecundity. The target of DILP8 appears to be *Lgr3*-Gal4 expressing neurons in the brain and ventral nerve cord, as well as a subset of the follicle cells found posteriorly in follicles with mature stage 14 eggs (see Figure 6). At this stage follicle cells in a similar position express Mmp2 and are critical for rupture and ovulation (48). We found some *Lgr3*-Gal4 expressing efferent neurons in the abdominal neuromeres that send axons to the oviduct muscle. Thus, DILP8 could act both in a paracrine fashion directly on follicle cells to induce follicle rupture and subsequent ovulation and also act systemically on neurons innervating the oviduct muscle that regulate ovulation. Since octopaminergic neurons innervating the oviduct have been implicated in regulation of ovulation (34, 48, 50, 51, 55, 56), and follicle cell maturation [see (57)], we tested whether octopaminergic neurons express the *Lgr3* receptor. However, we found no coexpression of *Lgr3-Gal4* and immunolabeling for TDC2, the biosynthetic enzyme of octopamine and tyramine, so it does not appear that DILP8 acts directly on octopaminergic neurons that regulate ovulation. The neurotransmitter of the efferent *Lgr3-Gal4* neurons that supply oviduct muscle, thus, remains to be identified.

It is remarkable that *dilp8*/DILP8 is restricted to follicle cells, as also indicated by available RNA expression data [FlyAtlas2 and ModEncode (20, 38)]. We do see *dilp8*-GFP expression also in cells of the hindgut and salivary glands, but could not find DILP8-immunolabeling in those tissues. Thus, adult expression is quite restricted, as is the case in larvae where DILP8 can be induced in imaginal disk, but not elsewhere in the organism (3, 4) (see also Supplementary Figure 1). In contrast, the DILP8 receptor *Lgr3* is expressed in a relatively large number of neurons in the brain and ventral nerve cord, both in larvae and adults (12, 13, 16, 21). So far only a specific set of *Lgr3-*Gal4 cells in the larval brain have been explored functionally. These two pairs of cells [growth coordinating Lgr3 (GCL) neurons] innervate the four neurons producing PTTH and thereby regulate ecdysone production in the prothoracic gland to produce a developmental delay (12, 13, 16). In adult flies, activation of sex-specific *Lgr3-*Gal4 neurons in the abdominal neuromeres inhibits female reproductive receptivity and fecundity (21). We did not analyze effects of *Lgr3* manipulations here, but suggest that this receptor in follicle cells and perhaps in a small set of efferent *Lgr3-*Gal4 neurons innervating oviduct muscle may be primarily responsible for the ovulation phenotype. It cannot be excluded that other *Lgr3*-Gal4 expressing neurons in the abdominal ganglia interact with octopaminergic neurons innervating the oviduct and thus indirectly contribute to the known octopamine effects on ovary maturation and ovulation (48, 50, 51, 55, 58, 59).

We noted effects of *dilp8* manipulations on resistance to starvation, which might suggest that the follicle cells signal systemically to regulate metabolism. Possibly this reflects feedback nutritional signaling from the ovaries to brain neurosecretory cells to ensure allocation of nutrients. There is no *Lgr3-*Gal4 expression in brain insulin-producing cells (IPCs) or other MNCs, and in adult flies we failed to demonstrate synaptic proximity between *Lgr3-*Gal4 neurons and IPCs using the GRASP technique. However, in larvae, a proximity between these neurons was seen (note that this technique alone does not reveal functional contacts). There are however, sets of *Lgr3-*Gal4 neurons with processes in pars intercerebralis where IPCs have dendrites (see Figures 2A–D). Thus, DILP8 might act on these *Lgr3*-Gal4 neurons and they may in turn signal to IPCs or other MNCs in a paracrine (non synaptic) fashion. Alternatively DILP8 might act directly on IPCs or other MNCs via the tyrosine kinase insulin receptor, dInR. It was shown that dInR could be captured by DILP8 in a ligand capture assay, possibly suggesting that DILP8 could bind to both Lgr3 and dInR (13).

Furthermore, we showed that *dilp8* mutants display increased resistance to desiccation, suggesting effects of DILP8 on water homeostasis. This could be mediated indirectly via action on *Lgr3*-Gal4 expressing neurons in the abdominal ganglia that produce diuretic or antidiuretic hormones. In larvae we detected *Lgr3* expression in leucokinin-producing neurosecretory cells (ABLKs) in abdominal ganglia, known to regulate water homeostasis in adult flies (46). These neurons also express the dInR both in larvae and adults (60). However, in adults the *Lgr3*-Gal4-leucokinin coexpression was not seen, but we found *Lgr3*-Gal4 expession in a small set of peripheral neurons that produce ion transport peptide, an antidiuretic hormone (32, 49). The functional role of these neurons in water homeostasis remains to be demonstrated and it would be interesting to determine whether DILP8 regulates their activity.

In summary, we found that *dilp8*/DILP8 is expressed in follicle cells of the ovaries, and that fecundity and starvation resistance (probably reflecting action on metabolism) are affected by manipulations of *dilp8*. We propose that DILP8 signaling might represent a feedback from ovaries to the brain to ensure allocation of nutrients for egg maturation and that DILP8 also constitutes a signal that aids in ovulation probably in concert with octopamine. Thus, DILP8 appears to act as a relaxin-like hormone in regulation of fecundity, suggesting conservation of an ancient function from insects to mammals.

## Data Availability Statement

The original contributions presented in the study are included in the article/Supplementary Material, further inquiries can be directed to the corresponding author/s.

## Author Contributions

SL: conceptualization, performed experiments, evaluated data, and wrote paper. DN: conceptualization, evaluated data, wrote paper, obtained funding, and supervised study. All authors contributed to the article and approved the submitted version.

## Conflict of Interest

The authors declare that the research was conducted in the absence of any commercial or financial relationships that could be construed as a potential conflict of interest.
